# Kinetic Study on the Isothermal and Nonisothermal Crystallization of Monoglyceride Organogels

**DOI:** 10.1155/2014/149753

**Published:** 2014-02-19

**Authors:** Zong Meng, Lijun Yang, Wenxin Geng, Yubo Yao, Xingguo Wang, Yuanfa Liu

**Affiliations:** ^1^State Key Laboratory of Food Science and Technology, Synergetic Innovation Center of Food Safety and Nutrition, School of Food Science and Technology, Jiangnan University, 1800 Lihu Road, Wuxi, Jiangsu 214122, China; ^2^Patent Examination Cooperation Center of the Patent Office, No. 3 Building, No. 11 Gaoliangqiaoxie Street, Haidian District, Beijing 100081, China

## Abstract

The isothermal and nonisothermal crystallization kinetics of monoglyceride (MAG) organogels were studied by pulsed nuclear magnetic resonance (*p*NMR) and differential scanning calorimetry (DSC), respectively. The Avrami equation was used to describe the isothermal crystallization kinetics and experimental data fitted the equation fairly well. Results showed that the crystal growth of MAG organogels was a rod-like growth of instantaneous nuclei at higher degrees of supercooling and a plate-like form with high nucleation rate at lower degrees of supercooling. The exothermic peak in nonisothermal DSC curves for the MAG organogels became wider and shifted to lower temperature when the cooling rate increased, and nonisothermal crystallization was analyzed by Mo equation. Results indicated that at the same crystallization time, to get a higher degree of relative crystallinity, a higher cooling rate was necessary. The activation energy of nonisothermal crystallization was calculated as 739.59 kJ/mol according to the Kissinger method. Therefore, as the results of the isothermal and nonisothermal crystallization kinetics for the MAG organogels obtained, the crystallization rate, crystal nucleation, and growth during the crystallization process could be preliminarily monitored through temperature and cooling rate regulation, which laid the foundation for the real industrial manufacture and application of the MAG organogels.

## 1. Introduction

Repeated consumption of a large number of saturated and trans fats in the solid fat products, such as shortenings, margarines, fat spreads, and chocolate, leads to a negative impact on human health by increasing the risk of metabolic syndrome and cardiovascular diseases (CVD) [1–3]. It is estimated that by replacing 5% of energy intake from saturated fats with that from carbohydrates or monounsaturated fats, the associated risk of CVD would reduce 22% and 37%, respectively [4]. So, new methods are required to produce solid fat alternatives.

In recent years, organogels have attracted much research attention, which can be defined as lipophilic liquid and solid mixtures of bulk liquid vegetable oil entrapped within a thermoreversible, three-dimensional gel network. A large number of various types of oleogelators, which should be of food-grade, economic, and efficient, used to structure liquid oil have been reported, such as triacylglycerides (TAGs) [5], diacylglycerides (DAGs), monoglycerides (MAGs) [6, 7], food-grade waxes [8, 9], protein walls [10], mixtures of *β*-sitosterol and *γ*-oryzanol [11, 12], mixtures of long-chain fatty acids and fatty alcohols [13], and mixtures of lecithin and sorbitan tristearate [14]. Among these gelators, saturated MAGs are regarded as one of those with a great potential for a wide range of application. Concentrated mixtures of liquid oils and MAGs can form semisolid, stable network structures upon cooling from the melted state, and the MAG/oil mixtures could be served as healthy substitutes of the margarine or butter without the attendant high levels of trans and saturated fatty acids. The MAG molecules can build a three-dimensional network structure by self-organization via noncovalent interactions, for example, van der Waals interactions, hydrogen bonding, *π*-stacking, and coordination bonds [15]. Physicochemical properties and crystallization behavior of MAG organogels have been investigated by several groups [16–19]. Da Pieve et al. used saturated MAGs to gelate cod liver oil; the results showed that shear processing during crystallization led to the formation of a weak gel network with a low oil binding capacity, while the opposite situation was obtained under static crystallization, and also demonstrated double effects on the oxidation of cod liver oil, which was ineffective on the first step of oxidation but hindered the formation of the secondary oxidation product [16, 17]. Ojijo et al. investigated the effects of MAG content, cooling rate, and shear on the temperature ramp, mechanical spectra, and hardness of olive oil/MAG gel networks; the results showed that the onset temperature of structure formation, final values of elastic modulus, and network hardness of the system all increased with the increasing content of MAGs [18]. Compared to the polarizing light microscopy, polarizing near-field scanning optical microscopy provided additional information on the structural organization of olive oil/MAG coagels at the micro- and submicron scale [19]. According to these studies, however, a fundamental question on how the organogels formed through structuring liquid vegetable oil on the molecular scale were connected with the their physicochemical properties on the macroscopic scale is still unclear. Moreover, to date, attempts have not yet been made regarding the comparative analysis of the isothermal and nonisothermal crystallization kinetics of organogels. Similar to the traditional fat products, the crystallization behavior of the system profoundly influences the final structure of organogels.

In the present research, the isothermal and nonisothermal crystallization kinetics of MAG organogels were examined systematically in a comparative study. The isothermal crystallization behavior is analyzed by pulsed nuclear magnetic resonance (*p*NMR), and the isothermal crystallization kinetic is described by the Avrami equation [20, 21]. While in the real industrial production and transportation of fat products the crystallization behavior usually occurs under the nonisothermal conditions (crystallization temperature change over time), the research of the nonisothermal crystallization process can guide the manufacture and application of related products. The nonisothermal crystallization behavior is analyzed using differential scanning calorimetry (DSC) [22], and the nonisothermal crystallization kinetic is investigated using the Mo equation.

## 2. Experimental Section

### 2.1. Materials

Dimodan HS K-A saturated MAGs (fatty acid composition: 1.20% C_14:0_, 59.63% C_16:0_, 38.48% C_18:0_, and 0.68% C_18:1_; melting point 67.05 ± 0.5°C), supplied by Danisco (Shanghai, China), were used due to their common application as an ingredient in the food industry. Canola oil (CaO) was generously provided by Kerry Specialty Fats Ltd. (Shanghai, China), which was chosen as the target lipid matrix to prepare the organogels, because it contains the lowest level of long-chain saturated fatty acids, such as 4.12% C_16:0_ and 1.81% C_18:0_, among common dietary fats and oils.

### 2.2. Preparation of MAG Organogels

Appropriate amount of CaO was weighed in a 100 mL beaker and heated on a magnetic stirrer heating plate to a temperature of 75 ± 5°C. Then 8% (w/w) of MAGs was added to the hot oil, and the mixture stirred until the MAGs were melted completely. The samples were then crystallized until reaching 20°C at a rate of 15°C min^−1^. The organogels were stored at 20°C under static conditions for 24 h to ensure that adequate time was given to anneal the network giving the maximum structure. All processes were duplicated.

### 2.3. *p*NMR

Solid fat content (SFC) was measured by *p*NMR using a AM4000 MQC NMR analyzer (Oxford Instruments, Abingdon, UK). The water bath based cooling used in the *p*NMR experiments also offered rapid cooling and accurate temperature control. Instrument was automatically calibrated using three standards (supplied by Oxford Instruments) containing 0%, 29.3%, and 69.7% solid. Samples were run in triplicate, and the values were averaged. The SFC of the samples was determined using the following thermal treatment: NMR tubes were filled with the melted sample (about 2.5 g), kept at 80°C for 30 min, and then placed in a thermostated water bath at 20, 25, 30, and 35°C, and SFC readings were obtained at appropriate time intervals.

### 2.4. DSC

Calorimetric analyses of the MAG organogels were made using a TA Q2000 DSC instrument (TA Instruments, New Castle, USA). Samples (*∼*0.8 mg) were hermetically sealed in an aluminum pan with an empty pan serving as a reference. The samples were heated to 70°C and held for 5 min to eliminate previous thermal history, and then the nonisothermal crystallization profiles were obtained by cooling to 10°C at the cooling rate of 1, 5, 10, and 20°C/min, respectively.

### 2.5. Avrami Model

*p*NMR data were fitted to the Avrami equation for isothermal crystallization. The Avrami equation can be used to quantify crystallization kinetics and give an indication of the nature of the crystallization process, including nucleation and growth [23]. It has the form

(1) $$\begin{array}{c} 1-X_{t}=\exp\left(-K_{t}t^{n}\right), \end{array}$$

where *X*_*t*_ is the relative degree of crystallinity at the time *t*, *K*_*t*_ is crystallization rate constant, which depends primarily on crystallization temperature, and *n*, the Avrami exponent, is a constant relating to the dimensionality of the transformation. The values of *K*_*t*_ and *n* are calculated from the intercept and slope, respectively, by the linear form of the Avrami equation as follows:

(2) $$\begin{array}{c} \ln\left[-\ln\left(1-X_{t}\right)\right]=n\ln t+\ln K_{t}. \end{array}$$

The numerical value of *K*_*t*_ is directly related to the half-time of crystallization, *t*_1/2_, and therefore the overall rate of crystallization [24], which is given by the following equation:

(3) $$\begin{array}{c} {\left(t_{1/2}\right)}^{n}=\frac{0.693}{K_{t}}. \end{array}$$

### 2.6. Mo Model

Considering that the nonisothermal is a nature of the crystallization process, Ozawa [25] had modified the Avrami equation by the way of assuming that the nonisothermal process is the result of an infinite number of small isothermal steps. According to the Ozawa theory, therefore, the relative crystallinity (*X*_*t*_) can be written as follows:

(4) $$\begin{array}{c} X_{t}=1-\exp\left(\frac{-K_{T}}{\varphi^{m}}\right), \end{array}$$

where *K*_*T*_ and *m* represent cooling crystallization temperature-dependent function and Ozawa exponent, respectively, *K*_*T*_ is referred to the crystallization rate (it indicates how fast crystallization occurs), *m* depends on the crystal dimension, and *φ* is the cooling rate (°C/min). However, the Ozawa analysis, when applied in certain fields (e.g., the polymer system), failed to describe their nonisothermal crystallization behavior [26].

For the purpose of finding a convenient approach to describe the nonisothermal crystallization process exactly, the research team of Mo combined the Avrami equation and Ozawa equation together, a method modified has been successfully applied to describe the nonisothermal crystallization kinetics of various polymers [26–31]. The final form of modified equation is shown as follows:

(5) $$\begin{array}{c} \text{lg}\;\varphi ={\text{lg}\left(\frac{K_{T}}{K_{t}}\right)}^{1/m}-\left(\frac{n}{m}\right)\text{lg}\;t, \end{array}$$

(6) $$\begin{array}{c} \text{lg}\;\varphi =\text{lg}\;F\left(T\right)-a\;\text{lg}\;t, \end{array}$$

where the parameter *F*(*T*) = (*K*_*T*_/*K*_*t*_)^1/*m*^ refers to the value of the cooling rate, which has to be chosen at unit crystallization time when the measured system amounts to a certain degree of crystallinity; *a* = (*n*/*m*) is the ratio of the Avrami exponent *n* to the Ozawa exponent *m*. According to (6), at a given degree of crystallinity, the plot of lg *φ* versus lg *t* will give a straight line, and the value of *F*(*T*) and *a* could be obtained by the intercept and the slope of the line, respectively.

In the study of nonisothermal crystallization using DSC, the energy released during the crystallization process appears to be a function of temperature. As a result, the relative crystallinity *X*(*T*), as a function of temperature, can be formulated as

(7) $$\begin{array}{c} X\left(T\right)=\frac{\int_{T_{0}}^{T}\left(dH_{c}/dT\right)dT}{\Delta H_{c}}, \end{array}$$

where *T*_0_ and *T* represent the onset and an arbitrary temperature, respectively, *dH*_*c*_ is the enthalpy of crystallization released during an infinitesimal temperature interval *dT*, and Δ*H*_*c*_ refers to the total enthalpy of crystallization at a certain cooling rate. During nonisothermal crystallization processes, the crystallization time domain can be transformed from the horizontal temperature scale; the crystallization time is calculated by the following equation:

(8) $$\begin{array}{c} t=\frac{\left|T_{0}-T\right|}{\varphi }, \end{array}$$

where *T* is the temperature at the crystallization time *t*, and *φ* is the cooling rate (°C/min).

### 2.7. Nonisothermal Crystallization Activation Energy

Detailed kinetic analysis can provide us more information with the thermal transition about the MAG organogels. Kissinger [32] developed a method to calculate the activation energy (Δ*E*) of the nonisothermal crystallization process, which can be formulated as:

(9) $$\begin{array}{c} \frac{d\left[\ln\left(\varphi /T_{p}^{2}\right)\right]}{d\left(1/T_{p}\right)}=-\frac{\Delta E}{R}, \end{array}$$

where *R* and *φ* represent the ideal gas constant (8.314 J·mol^−1^ k^−1^) and the cooling rate, respectively, and Δ*E* is the effective activation energy. ln⁡⁡(*φ*/*T*_*p*_^2^) versus 1/*T*_*p*_ can be obtained as a linear relationship, the slope of which shows the value of −Δ*E*/*R*. Then the value of Δ*E* can be derived from the slope.

## 3. Results and Discussion

### 3.1. Isothermal Crystallization Kinetics

**Figure 1:**
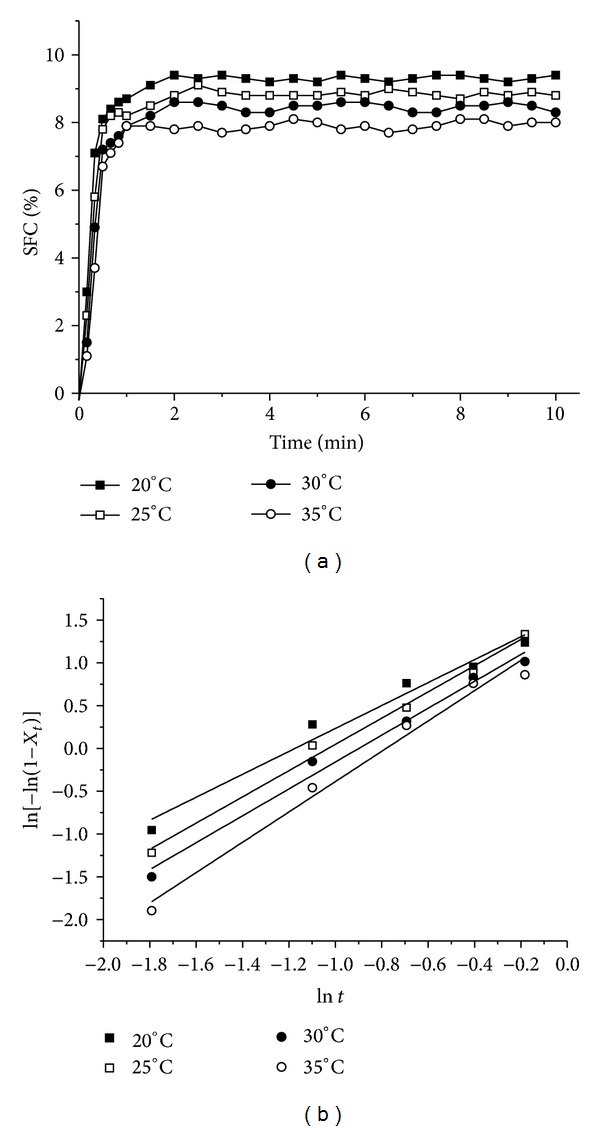
(a) SFC versus time of crystallization for MAG organogels at 20, 25, 30, and 35°C. (b) Plots of ln⁡[−ln⁡(1 − *X*_*t*_)] versus ln⁡*t* for isothermal crystallization of MAG organogels at 20, 25, 30, and 35°C, respectively.

The crystallization behavior of organogels profoundly influences the final structure of related application products and is intrinsically related to their macroscopic properties. Isothermal crystallization curves of MAG organogels at different temperatures are shown in Figure 1(a). Crystallization showed hyperbolic patterns against time, and an equilibrium value in SFC was clearly visible in all curves. The equilibrium SFC and the slope of crystallization curves decreased as the crystallization temperature increased from 20°C to 35°C, indicating that the crystallization rate slowed down gradually.

The Avrami model is the one most prevalently used to study the crystallization of fats and may be used to evaluate the crystallization kinetics and suggest the nature of crystal growth. To quantify differences in the crystallization behaviors of MAG organogels at different temperatures, respectively, the SFC crystallization kinetic data were fitted separately by Avrami equations. According to (2), the value of ln⁡[−ln⁡(1 − *X*_*t*_)] against ln⁡*t* gives a linear regression line of the MAG organogels at different crystallization temperature; the slope stands for *n* and the intercept for ln⁡*K*_*t*_, as shown in Figure 1(b). The equation fitted the data very well over the entire range of fractional crystallization shown in Table 1 (*R* ^2^ > 0.96 in all cases). The Avrami exponents (*n*), Avrami constants (*K*_*t*_), and half-times of crystallization (*t*_1/2_) determined from the curve fits are also shown in Table 1.

As previously introduced, crystallization temperature has a very strong influence on the crystallization rate constant (*K*_*t*_), which decreases with increasing temperature, indicating that crystallization proceeded more rapidly at a higher degree of supercooling (lower temperature). The declining *K*_*t*_ values also indicated a change in the nucleation and/or growth rate in MAG organogels under different temperatures noted above. Since *K*_*t*_ is a combined function of nucleation and growth as well as a strong function of temperature, the declines of which ineluctably induce changes in crystal morphology, such as crystal size and type [23]. Besides the parameter *K*_*t*_, the increase in *t*_1/2_ for the MAG organogels as a function of increasing crystallization temperature also reflected the decrease in *K*_*t*_ at higher temperatures. As shown in Table 1, the Avrami exponent *n* for MAG organogels was increased with increasing crystallization temperature. The change in *n* could indicate differences in crystal growth geometry and the type of nucleation because it is a function of the number of dimensions in which growth takes place; it reflects the details of fat crystal nucleation and growth mechanisms. Christian [33] has tabulated values for the Avrami exponent *n* for various types of nucleation and growth. For example, an *n* of 1 indicates rod-like growth from instantaneous nuclei; an *n* of 2 represents high nucleation rate and plate-like growth, where growth is primarily along two dimensions; an *n* of 3 indicates spherulitic growth from instantaneous nuclei, whereas an *n* of 4 represents heterogeneous nucleation and spherulitic growth from sporadic nuclei. For MAG organogels, an increase in Avrami exponents from *∼*1 to *∼*2 with increasing temperature is possibly also due to the type of fat crystal growth from rod-like form at higher degrees of supercooling to plate-like form at lower degrees of supercooling. Generally, low values of *n* and high values of *K*_*t*_ are associated with an increased rate of crystallization and a more instantaneous nucleation process with a shorter induction time, which, in turn, would yield smaller and more numerous crystals. Therefore, as the parameters of the isothermal crystallization kinetics for the MAG organogels are discussed above, the crystallization rate, crystal nucleation, and growth during the crystallization process could be preliminarily monitored through temperature regulation.

### 3.2. Nonisothermal Crystallization Kinetics

**Figure 2:**
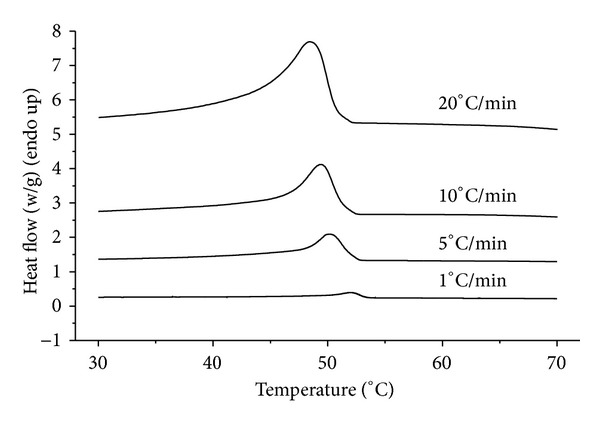
Nonisothermal melt crystallization exotherms for MAG organogels at four different cooling rates (1, 5, 10, and 20°C/min, resp.).

Figure 2 shows DSC curves of heat flow as a function of temperature at different cooling rates of 1, 5, 10, and 20°C/min for MAG organogels. It can be seen that the MAG organogels show a single peak from 70°C to 30°C under four different cooling rates mentionedabove. Clearly, when the cooling rate increased, the exothermic peak of the MAG organogels became wider and shifted to lower temperature, as would be expected for crystallization in a nucleation-controlled region. This was probably because of the fact that, under the lower cooling rate, MAG organogels crystallization could maintain a longer time at a higher temperature; the time was sufficient for the nucleation and crystal growth, so it could crystallize in a higher and narrower temperature range. In contrast, when the cooling rate was faster, the time was not sufficient for nucleation, and the crystal formation was incomplete, so that the crystallization temperature range was wider. Based on these curves, some kinetic data, such as the crystallization peak temperature (*T*_*p*_), the onset temperature (*T*_0_), and the enthalpy of crystallization (Δ*H*) for MAG organogels, can be taken and the values of these parameters are summarized in Table 2. It could be seen that all the crystallization parameters of MAG organogels were directly affected by the cooling rate. The values of *T*_0_ and *T*_*p*_ decreased while the Δ*H* increased gradually for the sample with increasing cooling rate.

**Figure 3:**
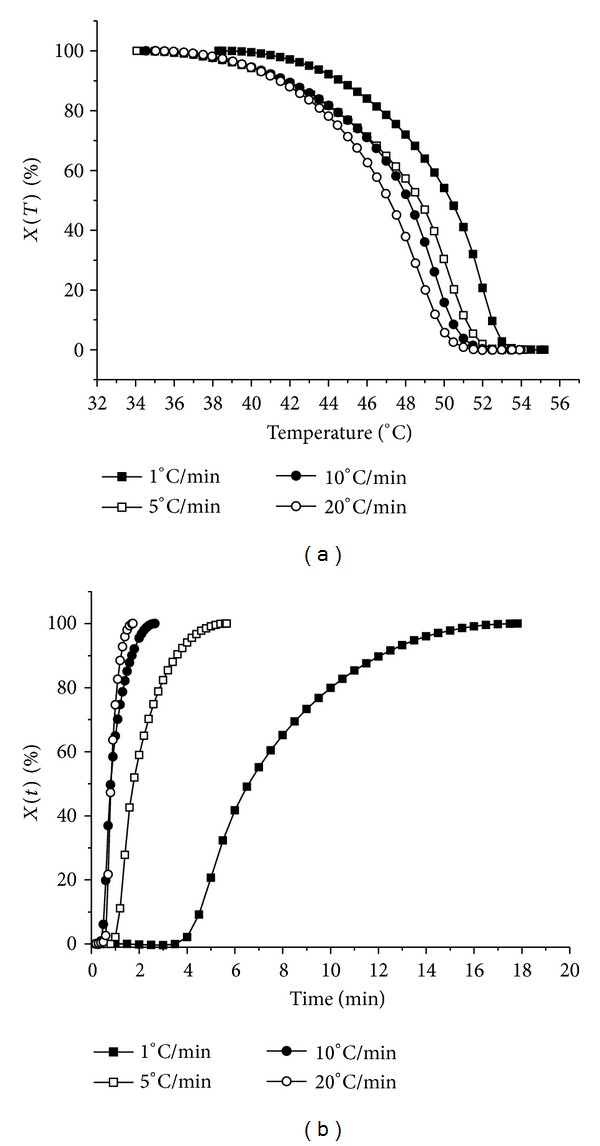
Plots of *X*(*T*) versus temperature (a) and plots of *X*(*t*) versus time (b) for crystallization of MAG organogels at four different cooling rates (1, 5, 10, and 20°C/min, resp.).

In order to obtain the kinetic information, the experimental data such as those shown in Figure 2 need to be converted to the relative crystallinity function of temperature *X*(*T*) using (7). The converted *X*(*T*) curves are illustrated in Figure 3(a). Apparently, all curves showed an approximately reversed sigmoidal shape, which showed a fast primary crystallization in the early stage and a slow secondary crystallization at the later stage. When the cooling rate increased, from the start to the end of the crystallization, the desired temperature range became broader, which was in accordance with the result obtained from Figure 2. The data can be further analyzed by converting the temperature scale of the *X*(*T*) function into the time scale, using (8), to get the relative crystallinity function of time *X*(*t*). The converted curves are shown in Figure 3(b). It could be seen that all these curves showed similar sigmoidal shapes. That is, there appeared an initial lag period where no sample was crystallized, followed by a period of rapid crystallization. Clearly, it is also noticed that the time for complete crystallization time increased as cooling rate decreased. Specifically, when the cooling rate reduced to 1°C/min, crystallization time was increased significantly.

### 3.3. Mo Analysis

**Figure 4:**
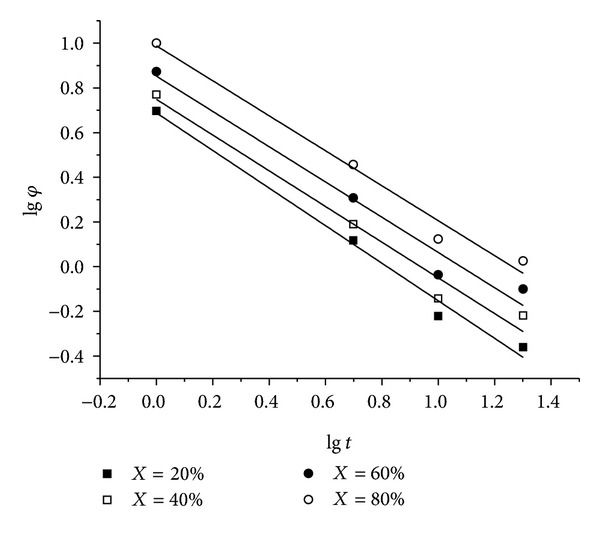
Plots of lg *φ* versus lg *t* for MAG organogels at the crystallinity of 20%, 40%, 60%, and 80%, respectively.

At a given degree of crystallinity, the plots of lg *φ* versus lg *t* for MAG organogels based on the Mo equation are shown in Figure 4, and the values of *F*(*T*) and *a* are obtained by the intercepts and the slopes of these lines, respectively, which are shown in Table 3. It showed that *F*(*T*) systematically increased from 4.875 to 9.727 with the rising relative degree of crystallinity. *F*(*T*) has a definite physical and practical significance, for a given degree of crystallinity. That is, the higher value of *F*(*T*), the higher cooling rate necessary within the unit crystallization time, indicating the difficulty of crystallization behavior. The parameter *a* shows only a small decrease with the increasing relative degree of crystallinity, ranging from 0.841 to 0.783, indicating that the ratio of *n* to *m* almost remained constant at different crystallinity.

**Figure 5:**
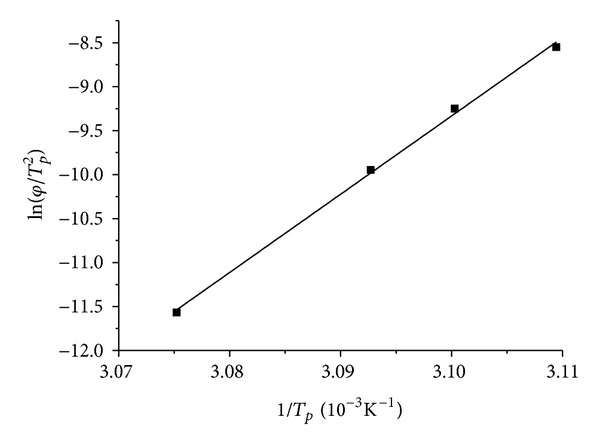
The plot of ln⁡(*φ*/*T*_*p*_^2^) versus 1/*T*_*p*_ for the MAG organogels.

Generally, the Kissinger activation energy characterizes the difficulty of the lipid crystallization process. The plot of ln⁡(*φ*/*T*_*p*_^2^) versus 1/*T*_*p*_ for the MAG organogels was shown in Figure 5, which fitted the Kissing equation quite well (*R* ^2^ > 0.99). The activation energy Δ*E* was obtained from the slope of the line and calculated as 739.59 kJ/mol.

## 4. Conclusions

In the present study, various classical models, namely, the Avrami, Mo, and Kissinger models, were applied to describe the isothermal and nonisothermal crystallization process of MAG organogels, respectively. The Avrami, the Mo, and the Kissinger models were found to describe the experimental data fairly well. The results of isothermal crystallization kinetics of the MAG organogels showed that the values of *n* increased from 1.340 to 1.772 with the increasing temperature range from 20°C to 35°C, indicating the transition of crystal growth from a rod-like form of instantaneous nuclei to a plate-like form with high nucleation rate. The exothermic peak in nonisothermal DSC curves for the MAG organogels became wider and shifted to lower temperature as the cooling rate increased. All curves of relative crystallinity versus crystallization temperature for the MAG organogels at the different cooling rate showed a reversed sigmoidal profile, which showed a fast primary crystallization in the early stage and a slow secondary crystallization at the later stage. The curves of relative crystallinity versus time showed similar sigmoidal shapes; namely, there appeared an initial lag period where no sample crystallized, followed by a period of rapid crystallization. The values of *F*(*T*) were increased from 4.875 to 9.727 with the increasing relative crystallization ranging between 20% and 80%, meaning that a higher cooling rate was required to reach a higher degree of relative crystallinity at the same crystallization time. The activation energy of nonisothermal crystallization was calculated as 739.59 kJ/mol according to the Kissinger method. Therefore, according to the results obtained in this study, the crystallization rate, crystal nucleation, and growth during the isothermal and nonisothermal crystallization process could be preliminarily monitored through temperature and cooling rate regulation, which are very useful for guiding the real industrial manufacture and application of the MAG organogels.

## Figures and Tables

**Table 1:** Avrami exponent (*n*), Avrami constant (*K*_*t*_), and half-time of crystallization (*t*_1/2_) for MAG organogels at 20, 25, 30, and 35°C, respectively.

Temperature (°C)	*n*	*K*_*t*_	*t*_1/2_ (min)	*R* ^2^
20	1.340	4.823	0.235	0.965
25	1.533	4.400	0.300	0.989
30	1.571	4.105	0.322	0.983
35	1.772	3.988	0.372	0.978

**Table 2:** Characteristic data of non-isothermal melt crystallization exotherms for MAG organogels.

*φ* (°C/min)	*T*_0_ (°C)	*T*_*p*_ (°C)	Δ*H*_*c*_ (J/g)
1	53.39	52.03	5.574
5	52.29	50.19	7.077
10	51.50	49.40	7.407
20	50.89	48.45	7.730

**Table 3:** Values of *F* (*T*) and *a* for crystallization of MAG organogels at the given degree of crystallinity.

*X* (%)	*F* (*T*)	*a*
20	4.875	0.841
40	5.598	0.799
60	7.129	0.789
80	9.727	0.783
